# Association Between Bone Mineral Density, Bone Turnover Markers, and Serum Cholesterol Levels in Type 2 Diabetes

**DOI:** 10.3389/fendo.2018.00646

**Published:** 2018-11-06

**Authors:** Yinqiu Yang, Guangwang Liu, Yao Zhang, Guiping Xu, Xilu Yi, Jing Liang, Chenhe Zhao, Jun Liang, Chao Ma, Yangli Ye, Mingxiang Yu, Xinhua Qu

**Affiliations:** ^1^Department of Endocrinology, Zhongshan Hospital, Fudan University, Shanghai, China; ^2^Department of Orthopaedics, Xuzhou Central Hospital, Xuzhou Clinical School of Xuzhou Medical University, The Affiliated XuZhou Hospital of Medical College of Southeast University, Xuzhou Clinical Medical College of Nanjing University of Chinese Medicine, Xuzhou, China; ^3^Department of Endocrinology, Department of Infectious Disease, Zhongshan Hospital, Fudan University, Shanghai, China; ^4^Department of Endocrinology, Zhongshan Hospital, Fudan University, Shanghai, China; ^5^VIP Clinical Department, Fujian Provincial Hospital, Fuzhou, China; ^6^Department of Endocrinology, Zhongshan Hospital, Songjiang Central Hospital, Fudan University, Shanghai, China; ^7^Department of Endocrinology, Xuzhou Central Hospital, Xuzhou Clinical School of Xuzhou Medical University, The Affiliated XuZhou Hospital of Medical College of Southeast University, Xuzhou Clinical Medical College of Nanjing University of Chinese Medicine, Xuzhou, China; ^8^Department of Bone and Joint Surgery, Renji Hospital, Shanghai Jiaotong University School of Medicine, Shanghai, China

**Keywords:** type 2 diabetes, osteoporosis, bone mineral density, bone turnover markers serum cholesterol, TC, HDL-C, LDL-C

## Abstract

**Purpose:** The association between bone mineral density (BMD), bone turnover markers, and serum cholesterol in healthy population has already been proved. However, in patients with type 2 diabetes mellitus (T2D), it has not been adequately analyzed. In this study, we investigated the correlation between BMD, bone turnover markers, and serum cholesterol levels in people with T2D.

**Methods:** We enrolled 1,040 men and 735 women with T2D from Zhongshan Hospital between October 2009 and January 2013. Their general condition, history of diseases and medication, serum markers, and BMD data were collected. We used logistic regression analysis to identify the association between serum cholesterol levels and BMD as well as bone turnover markers.

**Results:** In multivariate regression analysis, we observed that in men with T2D, high high-density lipoprotein-cholesterol and total cholesterol levels were significantly associated with low total lumbar, femur neck, and total hip BMD, while low-density lipoprotein-cholesterol level was only inversely associated with total lumbar and femur neck BMD. Total cholesterol and low-density lipoprotein-cholesterol levels were also negatively associated with osteocalcin, procollagen type I N-terminal propeptide, and β-crosslaps. In women with T2D, high-density lipoprotein-cholesterol level was observed to be negatively correlated with total lumbar, femur neck, and total hip BMD, while total cholesterol and low-density lipoprotein-cholesterol levels were only associated with BMD at the total lumbar. Furthermore, total cholesterol was also negatively associated with osteocalcin, procollagen type I N-terminal propeptide, and β-crosslaps; high-density lipoprotein-cholesterol was only related to osteocalcin and parathyroid hormone, while low-density lipoprotein-cholesterol was only related to β-crosslaps in women.

**Conclusion:** Our study suggests a significantly negative correlation between serum cholesterol levels and BMD in both men and women with T2D. The associations between serum cholesterol levels and bone turnover markers were also observed in T2D patients.

## Introduction

The prevalence of osteoporosis and low-energy fractures in the aging population is increasing, which may lead to disability, poor living quality, and even death (1). In patients with type 2 diabetes mellitus (T2D), the fracture risk is usually higher than that in the general population (2–4) and varies even after adjusting for age, duration of diabetes, antidiabetic drug usage, and body mass index (BMI) (5, 6). The risk factors for osteoporosis in the general population have been reported and analyzed in previous studies (7). However, because of metabolic disorders, the risk factors for osteoporosis in people with T2D may be different, and it is important to discuss them. According to current studies, several factors are regarded as risk factors for osteoporosis in people with diabetes. Afshinnia et al. (8) reported that in patients with diabetes, old age, low body weight, low serum calcium, and low-density lipoprotein cholesterol (LDL-C) levels were independently associated with lumbar spine osteoporosis (8). Chen et al. (9) observed that abnormal blood lipid, abnormal adipokine levels, and elevated inflammatory factor levels were independent risk factors for osteoporosis in patients with T2D (9).

In recent years, the association between serum lipid and bone metabolism has gained considerable interest; however, there is no general agreement regarding this subject yet. Retrospective studies conducted in postmenopausal women have reported a negative correlation between serum total cholesterol (TC), LDL-C levels, and bone mineral density (BMD) (10–12), while high-density lipoprotein-cholesterol (HDL-C) level was inversely associated with BMD in both men and women (11, 13–16). In addition, cholesterol-reducing medication, such as statins, was reported to have beneficial effects on BMD in most previous studies (17–19); this also suggests the negative association between serum cholesterol levels and BMD. However, positive association (20–22) as well as no correlation (23–27) has been reported in several studies. No study has analyzed the association between serum cholesterol level and BMD in a population with T2D. Only one study evaluating 229 American individuals that discussed the correlation between serum cholesterol levels and osteoporosis in people with diabetes discovered a direct correlation between LDL and lumbar spine osteoporosis (8).

The associations between serum cholesterol and bone turnover markers were also widely studied. Most studies found that TC, HDL-C, and LDL-C levels were negatively correlated with osteocalcin (OCN) in the general population (28, 29), while in patients with T2D, no association was reported (30, 31). Both procollagen type I N-terminal propeptide (PINP) and β-crosslaps (β-cTX) showed no relationship with serum cholesterol in healthy postmenopausal women as well as in T2D patients (12, 30). According to Ponda et al. in vitamin D repletion group, LDL-C was inversely correlated with serum parathyroid hormone (PTH) (32). However, another study found no association between PTH and serum cholesterol (33).

From the above, the association between BMD, bone turnover markers, and serum cholesterol remain controversial, and little research in T2D patients has been conducted. Therefore, we investigated the Chinese population with T2D, and aimed to clarify the association between serum cholesterol levels, including TC, HDL-C, and LDL-C, and BMD, at total lumbar, femur neck, and total hip. We analyzed men and women with T2D separately, as well as the linear and non-linear correlation.

## Materials and methods

### Study population

We conducted a hospital-based cross-sectional study. All participants were selected consecutively from the endocrinology department of Zhongshan Hospital between October 2009 and January 2013. All selected participants were >18 years old with T2D. T2D was diagnosed based on the Standards of Medical Care in Diabetes by the American Diabetes Association as follows: (a) hemoglobin A1c (HbA1c) ≥6.5%; or (b) fasting blood glucose (FBG) ≥7.0 mmol/L (no caloric intake for 8 h at least); or (c) 2-h blood glucose ≥11.1 mmol/L by oral glucose tolerance test (which uses glucose load containing the equivalent of 75 g anhydrous glucose dissolved in water); or (d) random blood glucose ≥11.1 mmol/L in patients with typical hyperglycemia symptoms or hyperglycemia crisis, which occurs in the absence of unequivocal hyperglycemia, results should be confirmed by repeat testing) (34). The exclusion criteria included (a) diagnosis of malignant tumor and severe heart, liver, or kidney diseases; (b) diagnosis of pituitary, thyroid, parathyroid, adrenal, and gonadal diseases; (c) long-term bedridden patients; (d) long history of using calcium, vitamin D, or other drugs that affect bone metabolism; and (e) patients lacking available information. Our final study sample comprised 1,776 diabetic patients (1,040 men and 735 women). The personal history and past medical history of each participant was obtained through individual questionnaires. This study was approved by the ethics committee of Zhongshan Hospital, Fudan University. All patients included in this retrospective observational study signed informed consent.

### Clinical assessment and health history

Trained doctors conducted overall physical examinations for each participant. Body weight and height were measured with light clothing and without shoes, and the minimum unit of measurement was 0.5 kg and 0.01 m, respectively. We calculated BMI by dividing weight (kg) by the square of height (m^2^). Blood pressure (systolic and diastolic) was measured in the sitting position and after at least 30 min of rest. We measured the blood pressure of each patient twice and obtained an average value to reduce errors. All other background information was voluntarily provided by the patients themselves or by their family members. Duration of diabetes was calculated in years, from the time the patient was diagnosed with T2D according to the patient's medical record, to the time we conducted blood tests and BMD measurements. The treatment of diabetes was classified into four categories: no treatment, insulin, oral medicine, and insulin and oral medicine. Smoking and drinking history were defined as never or ever. We also obtained the participants history of other diseases, family history, as well as history of trauma and operation.

### Biochemical parameters

Serum samples were collected at 6 a.m. after overnight fasting (8 h at least) and stored at room temperature for no more than 4 h before assay. Standard laboratory techniques were used to test glucose metabolism indices, including FBG and HbA1c; serum lipid metabolism indices, including TC, HDL-C, and LDL-C levels; and bone metabolism markers, including PTH, OCN, PINP, β-cTX, and 25-hydroxy-vitamin D [25(OH)D]. In addition, other laboratory markers such as hypersensitive C-reactive protein (hsCRP), blood creatinine (Cr), blood urea nitrogen (BUN), calcium (Ca), alanine aminotransferase (ALT), aspartate aminotransferase (AST), and alkaline phosphatase (ALP) levels were recorded.

### BMD measurement

We used dual-energy X-ray absorptiometry (Hologic-Discovery, USA) to detect the BMD of each patient at three sites: total lumbar, femur neck, and total hip.

### Statistical analysis

We separately performed analyses in men and women. We used mean ± standard deviation (SD) to describe continuous variables and used the number and proportion to describe categorical variables. We used chi-square tests for categorical variables, one-way ANOVA for normally distributed continuous variables, and the Kruskal–Wallis test for skewed continuous variables. We calculated the regression coefficient and corresponding 95% confidence intervals (CI) using unadjusted and multivariate adjusted logistic regression analyses for the associations between per SD in serum cholesterol level and BMD. The crude model was adjusted for no variables. The multivariate-adjusted model 1 was adjusted for age; diabetes duration (y); treatment of DM; smoking; drinking; and BMI. The multivariate-adjusted model 2 was further adjusted for age; diabetes duration (y); treatment of DM; smoking; drinking; BMI; cerebrovascular disease; kidney disease; family history of DM; diastolic blood pressure; FBG, mmol/L; Cr, μmol/L; BUN, mmol/L; Ca, mmol/L; ALT, U/L; AST, U/L; and ALP, U/L. The multivariate-adjusted model 3 was adjusted for age; diabetes duration (y); treatment of DM; smoking; drinking; BMI; cerebrovascular disease; kidney disease; family history of DM; diastolic blood pressure; FBG, mmol/L; Cr, μmol/L; BUN, mmol/L; Ca, mmol/L; ALT, U/L; AST, U/L; ALP, U/L; HbA1c, %; and hsCRP, mg/L. A two-sided *P*-value of <0.05 was considered to be statistically significant.

To examine the non-linear association between serum cholesterol level and osteoporotic fracture (logOR), we further applied a two-piecewise linear regression model using a smoothing function after adjusting for age; diabetes duration (y); treatment of DM; smoking; drinking; BMI; cerebrovascular disease; kidney disease; family history of DM; diastolic blood pressure; FBG, mmol/L; Cr, μmol/L; BUN, mmol/L; Ca, mmol/L; ALT, U/L; AST, U/L; ALP, U/L; PTH, pg/mL; OCN, ng/mL; PINP, ng/mL; β-cTX, pg/mL; and 25(OH)D, nmol/L. In addition, we conducted a log likelihood ratio test comparing the one-line linear regression model with the two-piecewise linear model.

To analyze the association between serum cholesterol levels and bone turnover markers, we used multivariate-adjusted model, adjusted for age; treatment of DM; diabetic duration; smoking; drinking; family history of DM; BMI; systolic blood pressure; diastolic blood pressure, FBG, mmol/L, Cr, umol/l, BUN, mmol/l, Ca, mmol/L; ALT, U/L; AST, U/L; and ALP, U/L.

Statistical analyses were performed using R packages (http://www.r-project.org) and Empower (R) (www.empowerstats.com, X&Y solutions Inc., Boston, MA).

## Results

### Basic characteristics

The patient characteristics are presented in Table 1. This retrospective study included 1776 patients with T2D with the mean age of 58.4 ± 13.3 years and BMI of 24.9 ± 3.7 kg/m^2^. The mean diabetic duration was 7.6 ± 7.0 years and mean FBG was 8.6 ± 3.1 mmol/L. The mean serum TC, HDL-C, and LDL-C levels of all participants were 4.6 ± 1.1, 1.1 ± 0.3, and 2.6 ± 0.9 mmol/L, respectively. The mean BMD at total lumbar, femur neck, and total hip were 1.0 ± 0.2, 0.8 ± 0.1, and 0.9 ± 0.1 g/cm^2^, respectively. Men were significantly younger than women (56.2 vs. 61.4 years, *P* < 0.001) and the diabetes duration was shorter (6.6 vs. 8.9, *P* < 0.001). The mean HbA1c was higher in men (9.5 vs. 9.1, *P* < 0.001), while mean β-cTX was higher in women (0.4 vs. 0.5, *P* = 0.012). The proportion of smoking or drinking history was significantly higher in men than in women (smoking: 42.4 vs. 2.2%; drinking: 20.9% vs. 1.2%, both *P* < 0.001).

**Table 1:** Patient characteristics, stratified by sex. Values are mean ± SD or n (%) unless otherwise specified.

	**Total patients (*n* = 1,776) (Mean ± SD or *N* %)**	**Male patients (*n* = 1,040) (Mean ± SD or *N* %)**	**Female patients (*n* = 736) (Mean ± SD or *N* %)**	***P*-value**
Age	58.355 ± 13.254	56.176 ± 13.611	61.433 ± 12.090	<0.001
Diabetic duration(y)	7.562 ± 6.997	6.612 ± 6.591	8.906 ± 7.330	<0.001
Systolic blood pressure	132.024 ± 16.814	130.835 ± 16.180	133.705 ± 17.545	<0.001
Diastolic blood pressure	81.042 ± 9.549	81.269 ± 9.473	80.720 ± 9.653	0.233
BMI	24.929 ± 3.720	24.883 ± 3.638	24.994 ± 3.835	0.542
**Laboratory findings**				
FBG, mmol/l	8.642 ± 3.053	8.705 ± 2.965	8.554 ± 3.174	0.308
HbA1C, %	9.310 ± 2.349	9.488 ± 2.375	9.059 ± 2.289	<0.001
hsCRP, mg/l	4.831 ± 10.824	4.698 ± 10.348	5.022 ± 11.478	0.551
TC, mmol/l	4.579 ± 1.081	4.582 ± 1.089	4.575 ± 1.069	0.891
HDL-C, mmol/l	1.113 ± 0.325	1.116 ± 0.329	1.110 ± 0.320	0.728
LDL-C, mmol/l	2.639 ± 0.914	2.643 ± 0.926	2.634 ± 0.896	0.842
Cr, umol/l	69.556 ± 26.392	69.642 ± 27.571	69.433 ± 24.651	0.870
BUN, mmol/l	5.899 ± 2.295	5.900 ± 2.283	5.896 ± 2.313	0.969
PTH, pg/ml	36.214 ± 14.546	36.193 ± 14.522	36.243 ± 14.592	0.945
OCN, ng/ml	13.942 ± 6.241	13.824 ± 6.039	14.113 ± 6.523	0.353
PINP, ng/ml	39.739 ± 19.623	39.196 ± 18.653	40.530 ± 20.945	0.178
β-cTX, pg/ml	0.447 ± 0.287	0.432 ± 0.268	0.469 ± 0.310	0.012
25(OH)D, nmol/l	35.130 ± 17.305	34.549 ± 17.248	35.974 ± 17.365	0.096
Ca, mmol/l	2.224 ± 0.114	2.224 ± 0.119	2.224 ± 0.108	0.977
ALT, U/L	26.929 ± 29.240	27.206 ± 28.079	26.539 ± 30.817	0.637
AST, U/L	23.451 ± 19.895	23.766 ± 20.996	23.007 ± 18.235	0.430
**BMD, g/cm**^2^				
Total lumbar	0.963 ± 0.160	0.963 ± 0.157	0.964 ± 0.164	0.902
Femur neck	0.756 ± 0.133	0.755 ± 0.134	0.756 ± 0.133	0.972
Total Hip	0.899 ± 0.141	0.899 ± 0.140	0.900 ± 0.142	0.923
**Treatment of diabetes**				0.043
No treatment	263 (14.809%)	175 (16.827%)	88 (11.957%)	
Insulin	464 (26.126%)	267 (25.673%)	197 (26.766%)	
Oral medicine	704 (39.640%)	401 (38.558%)	303 (41.168%)	
Insulin and OM	345 (19.426%)	197 (18.942%)	148 (20.109%)	
**Smoking**				<0.001
Never	1319 (74.268%)	599 (57.596%)	720 (97.826%)	
Current or ever	457 (25.732%)	441 (42.404%)	16 (2.174%)	
**Drinking**				<0.001
Never	1550 (87.275%)	823 (79.135%)	727 (98.777%)	
Current or ever	226 (12.725%)	217 (20.865%)	9 (1.223%)	
**Cerebrovascular disease**				0.710
No	1604 (90.315%)	937 (90.096%)	667 (90.625%)	
Yes	172 (9.685%)	103 (9.904%)	69 (9.375%)	
**Kidney disease**				0.069
No	1614 (90.878%)	956 (91.923%)	658 (89.402%)	
Yes	162 (9.122%)	84 (8.077%)	78 (10.598%)	
**Family history of diabetes**				0.052
No	1072 (60.360%)	608 (58.462%)	464 (63.043%)	
Yes	704 (39.640%)	432 (41.538%)	272 (36.957%)	

### Association between serum cholesterol levels and total lumbar BMD

In the univariate logistic regression model, serum cholesterol levels were significantly negatively associated with total lumbar BMD in both men and women with T2D. In men, a 1-SD increases in TC, HDL-C, and LDL-C levels were associated with 0.019-g/cm^2^ (*P* = 0.0003, 95% CI = 0.009–0.029), 0.031-g/cm^2^ (*P* < 0.0001, 95% CI = 0.021–0.042), and 0.017-g/cm^2^ (*P* = 0.0010, 95% CI = 0.007–0.028) decreases in total lumbar BMD, respectively. In women, a 1-SD increases in TC, HDL-C, and LDL-C levels correlated with total lumbar BMD decreases of 0.015 g/cm^2^ (*P* = 0.0336, 95% CI = 0.001–0.028), 0.027 g/cm^2^ (*P* = 0.0001, 95% CI = 0.014–0.040), and 0.014 g/cm^2^ (*P* = 0.0375, 95% CI = 0.001–0.028), respectively.

**Figure 1:**
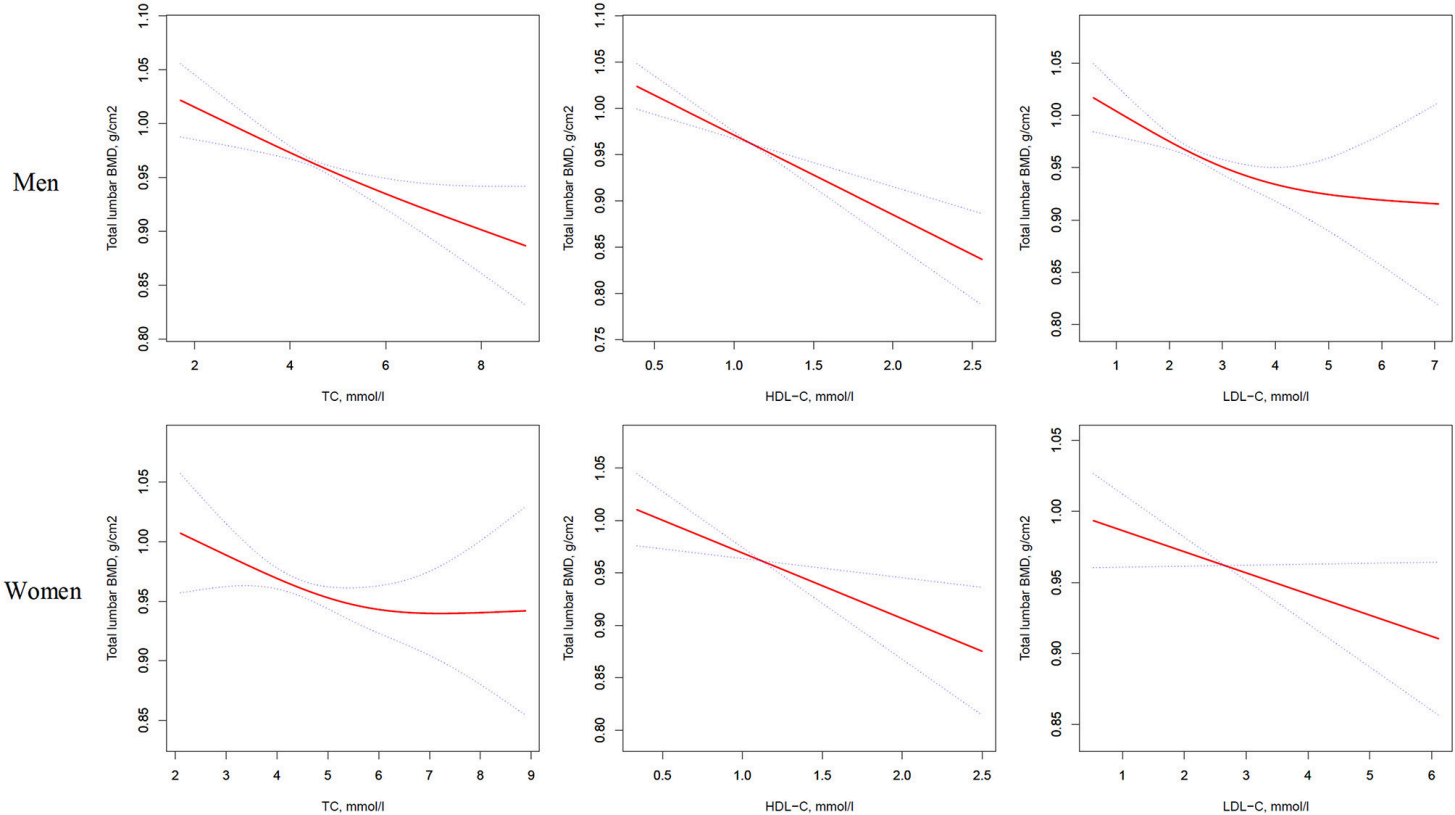
Multivariate adjusted smoothing spline plots of total lumbar BMD by TC, HDL-C, and LDL-C. Red dotted lines represent the spline plots of TC, HDL-C, and LDL-C and blue dotted lines represent the 95% CIs of the spline plots. Adjusted for age; diabetic duration(y); treatment of DM; smoking; drinking; BMI; cerebrovascular disease; kidney disease; family history of DM; diastolic blood pressure; FBG, unit; Cr, umol/l; BUN, mmol/l; Ca, unit; ALT, U/L; AST, U/L; ALP, U/L.

In addition, a similar negative association was observed in multivariate logistic regression analysis results (multivariate-adjusted model 3). In men, after multivariate adjustment, a 1-SD increases in TC, HDL-C, and LDL-C were associated with 0.022-g/cm^2^ (*P* = 0.0001, 95% CI = 0.011–0.034), 0.028-g/cm^2^ (*P* < 0.0001, 95% CI = 0.017–0.040), and 0.020-g/cm^2^ (*P* = 0.0002, 95% CI = 0.009–0.032) decreases, respectively in total lumbar BMD. In women, a 1-SD increase in TC, HDL-C, and LDL-C correlated with the total lumbar BMD decreases of 0.016 g/cm^2^ (*P* = 0.0336, 95% CI = 0.001–0.031), 0.020-g/cm^2^ (*P* = 0.0104, 95% CI = 0.005–0.035), and 0.018-g/cm^2^ (*P* = 0.0190, 95% CI = 0.003–0.032), respectively (Table 2 and Figure 1).

**Table 2:** Multivariate regression for effect of TC, HDL-C, and LDL-C on total lumbar BMD.

	**Male patients**		**Female patients**	
	**β(95%CI)**	***P***	**β(95%CI)**	***P***
**CRUDE MODEL**				
TC, mmol/l per SD	−0.019 (−0.029, −0.009)	0.0003	−0.015 (−0.028, −0.001)	0.0336
HDL-C, mmol/l per SD	−0.031 (−0.042, −0.021)	<0.0001	−0.027 (−0.040, −0.014)	0.0001
LDL-C, mmol/l per SD	−0.017 (−0.028, −0.007)	0.0010	−0.014 (−0.028, −0.001)	0.0375
**MULTIVARIATE-ADJUSTED MODEL 1**				
TC, mmol/l per SD	−0.021 (−0.031, −0.010)	0.0001	−0.015 (−0.029, −0.001)	0.0347
HDL-C, mmol/l per SD	−0.031 (−0.042, −0.021)	<0.0001	−0.028 (−0.042, −0.015)	0.0001
LDL-C, mmol/l per SD	−0.019 (−0.030, −0.008)	0.0004	−0.014 (−0.028, 0.000)	0.0565
**MULTIVARIATE-ADJUSTED MODEL 2**				
TC, mmol/l per SD	−0.021 (−0.032, −0.010)	0.0002	−0.013 (−0.028, 0.001)	0.0649
HDL-C, mmol/l per SD	−0.028 (−0.039, −0.017)	<0.0001	−0.020 (−0.035, −0.006)	0.0057
LDL-C, mmol/l per SD	−0.019 (−0.030, −0.008)	0.0006	−0.014 (−0.028, 0.001)	0.0616
**MULTIVARIATE-ADJUSTED MODEL 3**				
TC, mmol/l per SD	−0.022 (−0.034, −0.011)	0.0001	−0.016 (−0.031, −0.001)	0.0336
HDL-C, mmol/l per SD	−0.028 (−0.040, −0.017)	<0.0001	−0.020 (−0.035, −0.005)	0.0104
LDL-C, mmol/l per SD	−0.020 (−0.032, −0.009)	0.0007	−0.018 (−0.032, −0.003)	0.0190

*Crude Model adjust for: None Multivariate-Adjusted Model 1 adjust for: Age; Diabetic duration(y); Treatment of DM; Smoking; Drinking; BMI*.

*Multivariate-Adjusted Model 2 adjust for: Age; Diabetic duration(y); Treatment of DM; Smoking; Drinking; BMI; Cerebrovascular disease; Kidney disease; Family history of DM; Diastolic blood pressure; FBG, mmol/l; Cr, umol/l; BUN, mmol/l; Ca, mmol/l; ALT, U/L; AST, U/L; ALP, U/L*.

*Multivariate-Adjusted Model 3 adjust for: Age; Diabetic duration(y); Treatment of DM; Smoking; Drinking; BMI; Cerebrovascular disease; Kidney disease; Family history of DM; Diastolic blood pressure; FBG, mmol/l; Cr, umol/l; BUN, mmol/l; Ca, mmol/l; ALT, U/L; AST, U/L; ALP, U/L; HbA1C, %; hsCRP, mg/l*.

In addition, as shown in Table S1, on comparing the highest quartile with the lowest quartile of serum TC, HDL-C, and LDL-C levels in men with T2D; total lumbar BMD decreases of 0.046 g/cm^2^ (*P* = 0.00347, 95% CI = 0.015–0.076), 0.072 g/cm^2^ (*P* = 0.00001, 95% CI = 0.040–0.103), and 0.048 g/cm^2^ (*P* = 0.00203, 95% CI = 0.017–0.078), respectively were observed.

### Association between serum cholesterol levels and femur neck BMD

As shown in Table 3, univariate logistic regression analysis suggested that with a 1-SD increases in TC, HDL-C, and LDL-C levels in men, femur neck BMD decrease of 0.012 g/cm^2^ (*P* = 0.0074, 95% CI = 0.003–0.021), 0.027 g/cm^2^ (*P* < 0.0001, 95% CI = 0.018–0.036), and 0.011 g/cm^2^ (*P* = 0.0126, 95% CI = 0.002–0.020), respectively were observed. However, in women, a 1-SD increase in HDL-C level was associated with 0.022-g/cm^2^ decrease in femur neck BMD (*P* < 0.0001, 95% CI = 0.012–0.033).

**Table 3:** Multivariate Regression for Effect of TC, HDL-C, and LDL-C on femur neck BMD.

	**Male patients**		**Female patients**	
	**β(95%CI)**	***P***	**β(95%CI)**	***P***
**CRUDE MODEL**				
TC, mmol/l per SD	−0.012 (−0.021, −0.003)	0.0074	−0.005 (−0.016, 0.006)	0.4042
HDL-C, mmol/l per SD	−0.027 (−0.036, −0.018)	<0.0001	−0.022 (−0.033, −0.012)	<0.0001
LDL-C, mmol/l per SD	−0.011 (−0.020, −0.002)	0.0126	−0.006 (−0.017, 0.005)	0.2653
**MULTIVARIATE-ADJUSTED MODEL 1**				
TC, mmol/l per SD	−0.013 (−0.022, −0.004)	0.0049	−0.006 (−0.017, 0.005)	0.2816
HDL-C, mmol/l per SD	−0.028 (−0.037, −0.019)	<0.0001	−0.024 (−0.035, −0.013)	<0.0001
LDL-C, mmol/l per SD	−0.012 (−0.020, −0.003)	0.0122	−0.008 (−0.019, 0.004)	0.1909
**MULTIVARIATE-ADJUSTED MODEL 2**				
TC, mmol/l per SD	−0.012 (−0.022, −0.003)	0.0086	−0.007 (−0.018, 0.004)	0.2283
HDL-C, mmol/l per SD	−0.026 (−0.035, −0.016)	<0.0001	−0.017 (−0.029, −0.006)	0.0037
LDL-C, mmol/l per SD	−0.011 (−0.021, −0.002)	0.0150	−0.008 (−0.020, 0.003)	0.1470
**MULTIVARIATE-ADJUSTED MODEL 3**				
TC, mmol/l per SD	−0.014 (−0.024, −0.005)	0.0039	−0.008 (−0.020, 0.004)	0.1782
HDL-C, mmol/l per SD	−0.025 (−0.035, −0.016)	<0.0001	−0.018 (−0.030, −0.006)	0.0033
LDL-C, mmol/l per SD	−0.012 (−0.022, −0.002)	0.0160	−0.011 (−0.023, 0.001)	0.0624

*Crude Model adjust for: None Multivariate-Adjusted Model 1 adjust for: Age; Diabetic duration(y); Treatment of DM; Smoking; Drinking; BMI*.

*Multivariate-Adjusted Model 2 adjust for: Age; Diabetic duration(y); Treatment of DM; Smoking; Drinking; BMI; Cerebrovascular disease; Kidney disease; Family history of DM; Diastolic blood pressure; FBG, mmol/l; Cr, umol/l; BUN, mmol/l; Ca, mmol/l; ALT, U/L; AST, U/L; ALP, U/L*.

*Multivariate-Adjusted Model 3 adjust for: Age; Diabetic duration(y); Treatment of DM; Smoking; Drinking; BMI; Cerebrovascular disease; Kidney disease; Family history of DM; Diastolic blood pressure; FBG, mmol/l; Cr, umol/l; BUN, mmol/l; Ca, mmol/l; ALT, U/L; AST, U/L; ALP, U/L; HbA1C, %; hsCRP, mg/l*.

**Figure 2:**
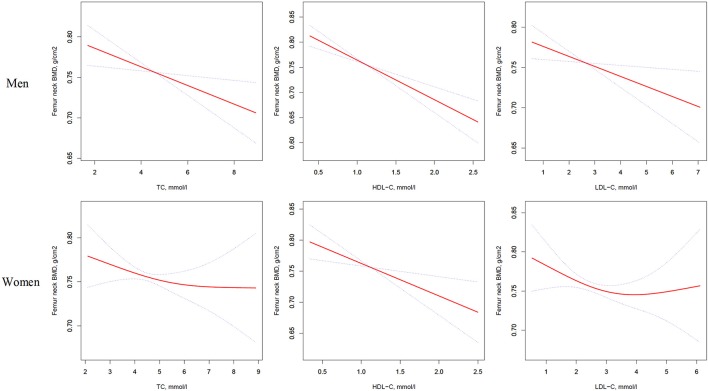
Multivariate adjusted smoothing spline plots of femur neck BMD by TC, HDL-C, and LDL-C. Red dotted lines represent the spline plots of TC, HDL-C, and LDL-C and blue dotted lines represent the 95% CIs of the spline plots. Adjusted for age; diabetic duration(y); treatment of DM; smoking; drinking; BMI; cerebrovascular disease; kidney disease; family history of DM; diastolic blood pressure; FBG, unit; Cr, umol/l; BUN, mmol/l; Ca, unit; ALT, U/L; AST, U/L; ALP, U/L.

In multivariate regression analyses with 1-SD increases in TC, HDL-C, and LDL-C levels in men, femur neck BMD decreases of 0.014 g/cm^2^ (*P* = 0.0039, 95% CI = 0.005–0.024), 0.025 g/cm^2^ (*P* < 0.0001, 95% CI = 0.016–0.035), and 0.012 g/cm^2^ (*P* = 0.0160, 95% CI = 0.002–0.022), respectively were observed. In women, a 1-SD increase in only HDL-C level was associated with a 0.018-g/cm^2^ (*P* = 0.0033, 95% CI = 0.006–0.030) decrease in femur neck BMD, and no association between TC and LDL-C levels and femur neck BMD was observed in our study (Table 3 and Figure 2).

Table S2 summarizes that on comparing the highest HDL-C level group to the lowest, total hip BMD in men with T2D decreased by 0.065 g/cm^2^ (*P* < 0.00001, 95% CI = 0.038–0.092) while that in women with T2D decreased by 0.041 g/cm^2^ (*P* = 0.01493, 95% CI = 0.008–0.074). In addition, in women with T2D, the highest LDL-C level group 0.033 g/cm^2^ (*P* = 0.03879, 95% CI = 0.002–0.064) lower femur neck BMD compared with the lowest quartile groups.

### Association between serum cholesterol levels and total hip BMD

**Figure 3:**
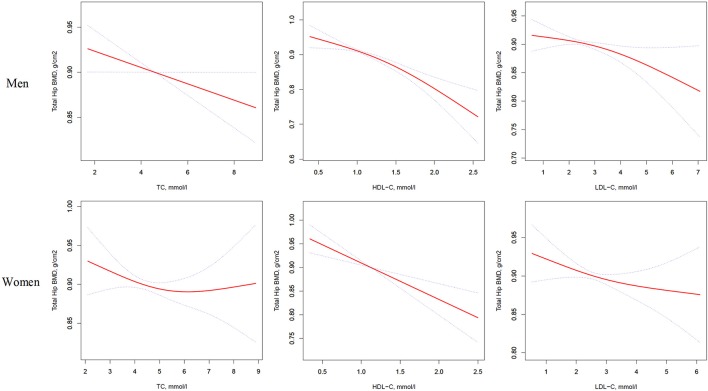
Multivariate adjusted smoothing spline plots of total hip BMD by TC, HDL-C, and LDL-C. Red dotted lines represent the spline plots of TC, HDL-C, and LDL-C and blue dotted lines represent the 95% CIs of the spline plots. Adjusted for age; diabetic duration(y); treatment of DM; smoking; drinking; BMI; cerebrovascular disease; kidney disease; family history of DM; diastolic blood pressure; FBG, unit; Cr, umol/l; BUN, mmol/l; Ca, unit; ALT, U/L; AST, U/L; ALP, U/L.

Table 4 and Figure 3 present the association between serum cholesterol levels and total hip BMD by the logistic regression model and two-piecewise linear regression model. In the crude model with a 1-SD increase in HDL-C and LDL-C levels, total hip BMD decreases of 0.033 g/cm^2^ (*P* < 0.0001, 95% CI = 0.023–0.042) in men and 0.030 g/cm^2^ (*P* < 0.0001, 95% CI = 0.04–0.019) in women were observed, while a 1-SD increase in LDL-C level was only found to be associated with 0.011-g/cm^2^ total hip BMD decrease in men (*P* = 0.0271, 95% CI = 0.001–0.020).

**Table 4:** Multivariate Regression for Effect of TC, HDL-C, and LDL-C on total hip BMD.

	**Male patients**		**Female patients**	
	**β(95%CI)**	***P***	**β(95%CI)**	***P***
**CRUDE MODEL**				
TC, mmol/l per SD	−0.009 (−0.018, 0.000)	0.0581	−0.005 (−0.016, 0.006)	0.3827
HDL-C, mmol/l per SD	−0.033 (−0.042, −0.023)	<0.0001	−0.030 (−0.041, −0.019)	<0.0001
LDL-C, mmol/l per SD	−0.011 (−0.020, −0.001)	0.0271	−0.007 (−0.019, 0.004)	0.2180
**MULTIVARIATE-ADJUSTED MODEL 1**				
TC, mmol/l per SD	−0.010 (−0.020, −0.001)	0.0375	−0.007 (−0.019, 0.005)	0.2859
HDL-C, mmol/l per SD	−0.034 (−0.043, −0.025)	<0.0001	−0.032 (−0.043, −0.020)	<0.0001
LDL-C, mmol/l per SD	−0.011 (−0.021, −0.002)	0.0218	−0.009 (−0.021, 0.004)	0.1640
**MULTIVARIATE-ADJUSTED MODEL 2**				
TC, mmol/l per SD	−0.010 (−0.020, 0.000)	0.0486	−0.007 (−0.019, 0.005)	0.2749
HDL-C, mmol/l per SD	−0.031 (−0.040, −0.021)	<0.0001	−0.025 (−0.037, −0.013)	0.0001
LDL-C, mmol/l per SD	−0.011 (−0.020, −0.001)	0.0286	−0.009 (−0.022, 0.003)	0.1310
**MULTIVARIATE-ADJUSTED MODEL 3**				
TC, mmol/l per SD	−0.011 (−0.021, 0.000)	0.0403	−0.008 (−0.021, 0.004)	0.1914
HDL-C, mmol/l per SD	−0.029 (−0.039, −0.019)	<0.0001	−0.028 (−0.041, −0.015)	<0.0001
LDL-C, mmol/l per SD	−0.010 (−0.020, 0.000)	0.0575	−0.013 (−0.026, 0.000)	0.0494

*Crude Model adjust for: None Multivariate-Adjusted Model 1 adjust for: Age; Diabetic duration(y); Treatment of DM; Smoking; Drinking; BMI*.

*Multivariate-Adjusted Model 2 adjust for: Age; Diabetic duration(y); Treatment of DM; Smoking; Drinking; BMI; Cerebrovascular disease; Kidney disease; Family history of DM; Diastolic blood pressure; FBG, mmol/l; Cr, umol/l; BUN, mmol/l; Ca, mmol/l; ALT, U/L; AST, U/L; ALP, U/L*.

*Multivariate-Adjusted Model 3 adjust for: Age; Diabetic duration(y); Treatment of DM; Smoking; Drinking; BMI; Cerebrovascular disease; Kidney disease; Family history of DM; Diastolic blood pressure; FBG, mmol/l; Cr, umol/l; BUN, mmol/l; Ca, mmol/l; ALT, U/L; AST, U/L; ALP, U/L; HbA1C, %; hsCRP, mg/l*.

After multivariate adjustment, a 1-SD increase in HDL-C level was found to decrease total hip BMD by 0.029 g/cm^2^ (*P* < 0.0001, 95% CI = 0.019–0.039) in men and 0.028 g/cm^2^ (*P* < 0.0001, 95% CI = 0.015–0.041) in women. The association between TC level and total hip BMD was observed only in men (β = −0.011, 95% CI = −0.000 to −0.021, *P* = 0.0403); and LDL-C level was not associated with total hip BMD in both sexes.

In Table S3, on comparing the highest HDL-C level group to the lowest, total hip BMD in men with T2D decreased by 0.069 g/cm^2^ (*P* < 0.00001, 95% CI = 0.041–0.096) while that in women with T2D decreased by 0.062 g/cm^2^ (*P* = 0.00064, 95% CI = 0.027–0.097).

For comparison, a longitudinal cohort study reported that in the late perimenopausal women, BMD declined substantially with an average loss of 0.018 and 0.010 g/cm^2^/yr from the spine and hip, respectively, and in the postmenopausal women, rates of loss from the spine and hip were 0.022 and 0.013 g/cm^2^/yr, respectively (*P* < 0.001 for all) (35). Therefore, there are clinical implications to the effects of serum cholesterol levels on BMD in our study.

### Association between serum cholesterol levels and bone turnover markers

#### OCN

In men with T2D, multivariate regression analysis showed that with values above the inflection point (TC > 0.79 mmol/L; HDL-C > 0.71 mmol/L), a 1-SD increase in TC was associated with 1.59 ng/mL (*P* = 0.0060, 95% CI = 0.46–2.72) decrease in OCN, while per mmol/L increase in HDL-C was associated with 1.95 ng/mL increase in OCN (*P* = 0.0034, 95% CI = 0.65–3.24). Moreover, when LDL-C was no more than 3.00 mmol/L, with per mmol/L increase in LDL-C, OCN increased 0.98 ng/mL (*P* = 0.0088, 95% CI = 0.25–1.17), whereas when and LDL-C increased above 3.00 mmol/L, with per mmol/L increase in LDL-C, OCN decreased 1.20 ng/mL (*P* = 0.0158, 95% CI = 0.23–2.17).

In women with T2D, when HDL-C was more than 1.18 mmol/L, with 1 mmol/L increase in HDL-C, OCN increased by 5.49 ng/mL (*P* = 0.008, 95% CI = 2.30–8.68). While TC and LDL-C was not associated with OCN (Table 5).

**Table 5:** Multivariate Regression for Effect of TC, HDL-C, and LDL-C on OCN.

	**Linear regression**		**Break point (K)**	<**K**		> **K**	
	**β (95%CI)**	***p***		**β (95%CI)**	***p***	**β (95%CI)**	***p***
**OCN(MALE)**							
TC, mmol/l per SD	−0.05 (−0.36, 0.46)	0.8068	0.79	0.71 (0.12, 1.29)	0.0185	−1.59 (−2.72, −0.46)	0.0060
LDL-C, mmol/l	0.10 (−0.34, 0.55)	0.6481	3.00	0.98 (0.25, 1.71)	0.0088	−1.20 (-2.17, −0.23)	0.0158
HDL-C, mmol/l	1.70 (0.46, 2.93)	0.0075	0.71	−8.23(−23.83, 7.37)	0.3013	1.95 (0.65, 3.24)	0.0034
**OCN(FEMALE)**							
TC, mmol/l per SD	0.39 (−0.16, 0.94)	0.1617	1.23	1.00 (0.27, 1.73)	0.0072	−1.76 (-3.55, 0.02)	0.0536
LDL-C, mmol/l	0.15 (−0.47, 0.76)	0.6389	4.24	0.46 (−0.26, 1.18)	0.2089	−2.47 (−5.66, 0.73)	0.1309
HDL-C, mmol/l	2.79 (1.09, 4.49)	0.0013	1.18	0.03 (−3.21, 3.28)	0.9832	5.49 (2.30, 8.68)	0.0008

*Multivariate-Adjusted Model adjust for: Age; Treatment of DM; Diabetic duration(y); Smoking; Drinking; Family history of DM; BMI; Systolic blood pressure; Diastolic blood pressure; FBG, mmol/l; Cr, umol/l; BUN, mmol/l; Ca, mmol/l; ALT, U/L; AST, U/L; ALP, U/L*.

#### PINP

As shown in Table 6, in multivariate regression analysis, we found that when TC was above 0.72 mmol/L, a 1-SD increase in TC in men was related with 4.62 ng/ml decrease in PINP (*P* = 0.0061, 95% CI = 1.33–7.92). Only when LDL-C was >2.77 mmol/L, with per mmol/L increase in LDL-C, PINP decreased by 4.02 ng/ml in men with T2D (*P* = 0.0023, 95% CI = 1.45–6.59).

**Table 6:** Multivariate Regression for Effect of TC, HDL-C, and LDL-C on PINP.

	**Linear regression**		**Break point (K)**	<**K**		> **K**	
	**β (95%CI)**	***p***		**β (95%CI)**	***p***	**β (95%CI)**	***p***
**PINP(MALE)**							
TC, mmol/l per SD	−1.09 (−2.34, 0.16)	0.0884	0.72	0.49 (−1.36, 2.35)	0.6024	−4.62 (−7.92, −1.33)	0.0061
LDL-C, mmol/l	−0.75 (−2.11, 0.61)	0.2784	2.77	2.52 (−0.05, 5.10)	0.0554	−4.02 (−6.59, −1.45)	0.0023
HDL-C, mmol/l	2.74 (−1.08, 6.55)	0.1599	0.94	−3.18(−18.47, 12.10)	0.6833	3.84 (−0.87, 8.55)	0.1103
**PINP(FEMALE)**							
TC, mmol/l per SD	0.06 (−1.73, 1.84)	0.9499	1.21	2.00 (−0.38, 4.37)	0.1008	−6.60 (−12.31, −0.88)	0.0241
LDL-C, mmol/l	−0.41 (−1.58, 2.40)	0.6866	4.23	1.58 (−0.75, 3.91)	0.1852	−9.17 (−19.39, 1.06)	0.0795
HDL-C, mmol/l	3.05 (−2.64, 8.74)	0.2945	1.71	5.21 (−1.28, 11.69)	0.1164	−23.82 (−70.50, 12.85)	0.1759

*Multivariate-Adjusted Model adjust for: Age; Treatment of DM; Diabetic duration(y); Smoking; Drinking; Family history of DM; BMI; Systolic blood pressure; Diastolic blood pressure; FBG, mmol/l; Cr, umol/l; BUN, mmol/l; Ca, mmol/l; ALT, U/L; AST, U/L; ALP, U/L*.

However, in women with T2D, only TC level was found to be related with PINP. When TC was >1.21 mmol/L, 1-SD increase in TC was correlated with 6.60 ng/ml decrease in PINP (*P* = 0.0241, 95% CI = 0.88–12.31).

#### β-CTX

Table 7 shows the correlation between serum cholesterol levels and β-cTX after multivariate regression analysis. In men with T2D, when TC was >0.76 mmol/L, a 1-SD increase in TC was correlated with 0.06 pg/ml decrease in β-cTX (*P* = 0.0028, 95% CI = 0.02–0.10), while 1 mmol/L increase in LDL-C was correlated with 0.09 pg/ml decrease in β-cTX (*P* = 0.0020, 95% CI = 0.03–0.115) only if LDL-C was >3.47 mmol/L. When LDL-C was < 3.47 mmol/L, with per mmol/L increase in LDL-C, β-cTX increased by 0.04 ng/ml (*P* = 0.0072, 95% CI = 0.01–0.06).

**Table 7:** Multivariate Regression for Effect of TC, HDL-C, and LDL-C on β-CTX.

	**Linear regression**		**Break point (K)**	< **K**		> **K**	
	**β (95%CI)**	***p***		**β (95%CI)**	***p***	**β (95%CI)**	***p***
β**-CTX(MALE)**							
TC, mmol/l per SD	−0.01 (−0.02, 0.01)	0.4646	0.52	0.03 (−0.00, 0.06)	0.0637	−0.06 (−0.10, −0.02)	0.0028
LDL-C, mmol/l	0.00 (−0.01, 0.02)	0.6381	3.47	0.04 (0.01, 0.06)	0.0072	−0.09 (−0.15, −0.03)	0.0020
HDL-C, mmol/l	0.04 (−0.02, 0.09)	0.1878	0.70	−0.61 (−1.34, 0.12)	0.1099	0.05 (−0.01, 0.11)	0.0769
β**-CTX(FEMALE)**							
TC, mmol/l per SD	0.00 (−0.02, 0.03)	0.7921	1.16	0.03 (−0.00, 0.07)	0.0575	−0.09 (−0.17, −0.01)	0.0210
LDL-C, mmol/l	0.01 (−0.02, 0.04)	0.5091	4.12	0.03 (−0.00, 0.06)	0.0814	−0.13 (−0.26, −0.00)	0.0481
HDL-C, mmol/l	0.06 (−0.02, 0.14)	0.1350	1.42	−0.01 (−0.12, 0.11)	0.9224	0.26 (0.01, 0.51)	0.0433

*Multivariate-Adjusted Model adjust for: Age; Treatment of DM; Diabetic duration(y); Smoking; Drinking; Family history of DM; BMI; Systolic blood pressure; Diastolic blood pressure; FBG, mmol/l; Cr, umol/l; BUN, mmol/l; Ca, mmol/l; ALT, U/L; AST, U/L; ALP, U/L*.

In women with T2D, with values above the inflection point (TC > 1.19 mmol/L; LDL-C > 3.94 mmol/L and HDL-C >1.42 mmol/L), with 1-SD increase in TC, β-cTX decreased by 0.09 pg/mL (*P* = 0.0210, 95% CI = 0.01–0.17); with per mmol/L increase in LDL-C, β-cTX decreased by 0.13 pg/mL (*P* = 0.0481, 95% CI = 0.00–0.26) and with per mmol/L increase in HDL-C, β-cTX increased by 0.26 pg/mL (*P* = 0.0433, 95% CI = 0.01–0.51).

#### PTH

In Table 8, in multivariate regression analysis, we found positive association between TC, HDL-C level and PTH in men with T2D. When TC was <0.45 mmol/L, a 1-SD increase in TC was associated with 1.80 pg/mL (*P* = 0.0385, 95% CI = 0.10–3.50) decrease in PTH, and when HDL-C was >0.77 mmol/L, per mmol/L increase in HDL-C was associated with 3.71 pg/mL (*P* = 0.0348, 95% CI = 0.27–7.15) decrease in PTH.

**Table 8:** Multivariate Regression for Effect of TC, HDL-C, and LDL-C on PTH.

	**Linear regression**		**Break point (K)**	<**K**		>**K**	
	**β (95%CI)**	***p***		**β (95%CI)**	***p***	**β (95%CI)**	***p***
**PTH(MALE)**							
TC, mmol/l per SD	1.07 (0.03, 2.10)	0.0439	0.45	1.80 (0.10,3.50)	0.0385	−0.04(−2.33,2.25)	0.9725
LDL-C, mmol/l	−0.68 (−0.45, 1.80)	0.2404	4.13	0.50 (−0.79, 1.78)	0.4484	2.28 (−3.36, 7.92)	0.4285
HDL-C, mmol/l	2.90 (−0.29, 6.09)	0.0751	0.77	−14.77(−42.97, 13.43)	0.3049	3.71 (0.27, 7.15)	0.0348
**PTH(FEMALE)**							
TC, mmol/l per SD	1.41 (0.14, 2.67)	0.0297	0.98	2.74 (0.96,4.53)	0.0027	−2.08(−5.16,1.46)	0.2499
LDL-C, mmol/l	0.61 (−0.81, 2.02)	0.4022	4.23	1.57 (−0.09, 3.24)	0.0643	−7.24 (−14.55, 0.06)	0.0524
HDL-C, mmol/l	5.43 (1.41, 9.44)	0.0084	1.72	8.72 (4.15, 13.29)	0.0002	−32.47(−58.47, −6.48)	0.0147

*Multivariate-Adjusted Model adjust for: Age; Treatment of DM; Diabetic duration(y); Smoking; Drinking; Family history of DM; BMI; Systolic blood pressure; Diastolic blood pressure; FBG, mmol/l; Cr, umol/l; BUN, mmol/l; Ca, mmol/l; ALT, U/L; AST, U/L; ALP, U/L*.

In women group, when HDL-C was <1.72 mmol/L, with 1 mmol/L increase in HDL-C, PTH increased by 8.72 pg/ml (*P* = 0.0002, 95% CI = 4.15–13.29) whereas, when HDL-C was > 1.72 mmol/L, 1 mmol/L increase in HDL-C was associated with 32.47 pg/ml decrease in PTH (*P* = 0.0147, 95% CI = 6.48–58.47). While a 1-SD increase in TC was associated with 2.74 pg/mL (*P* = 0.0027, 95% CI = 0.96–4.53) decrease in PTH only if TC was < 0.98 mmol/L.

## Discussion

To the best of our knowledge, our study was the first to report an inverse correlation between serum cholesterol levels and BMD in an Asian population with T2D. After multivariate-adjusted analyses, we observed a significantly negative association between HDL-C level and BMD at the total lumbar, femur neck, and total hip in both male and female patients with T2D. Moreover, in men with T2D, TC level was significantly negatively correlated with total lumbar BMD, femur neck BMD, and total hip BMD, while LDL-C level was only inversely related to the total lumbar BMD and the femur neck BMD. In women with T2D, TC, and LDL-C levels were found to be negatively related to the total lumbar BMD only.

Although controversial, the association between serum cholesterol and BMD has been widely studied in the general population; however, no such studies have been conducted on people with T2D. Only one study conducted in the American population with T2D reported that low LDL level was independently associated with lumbar spine osteoporosis, after adjusting for sex; race; ethnicity; and use of statins, plasma glucose, and other lipoproteins. Several studies have reported that the correlation between serum cholesterol and BMD may be affected by race. Therefore, our analysis of the effect of serum cholesterol on BMD in Asian T2D patients is of great significance.

Our study observed a significantly negative association between HDL-C level and BMD in patients with T2D. Makovey et al. (11) reported that BMD values were significantly lower in postmenopausal women with higher HDL-C levels in the general population, while Kuipers et al. (16) and Choi et al. (36) reported that a higher HDL-C level was associated with lower BMD in men. Adami et al. (13) and Buizert et al. (15) reported a negative relationship between HDL-C level and BMD in both healthy men and women. However, in studies conducted in both men (36) and women (26), HDL-C level was reported to be positively correlated with BMD, and several studies have reported no association between HDL-C level and BMD in postmenopausal women (27).

In our study, TC level was inversely associated with BMD at three sites in men with T2D, and in women, TC level was also inversely associated with total lumbar and femur neck BMD. According to Cui et al. (10), Makovey et al. (11), and Choi et al. (36), serum TC levels were inversely correlated with BMD in both pre- and postmenopausal women in the general population, and Garg et al. (37) observed that TC was weakly negatively associated with BMD in the Indian population in both sexes. In addition, TC was considered an independent risk factor for osteoporosis in some studies (38), which also suggested negative correlation with BMD. However, there are some controversies. Adami et al. (13) revealed that the relationship between TC and BMD was positive in healthy men and women. In addition, some studies reported no association between TC and BMD in postmenopausal women (26, 27) and the general population in both sexes (15, 23, 24).

Furthermore, our study demonstrated that LDL-C level was inversely correlated with BMD at total lumbar and femur neck in men with T2D, and in women, it was negatively associated with total lumber BMD. Similar inverse correlation between LDL-C level and BMD was reported in postmenopausal women (10–12), as well as in the general Asian population (36, 37) without T2D. However, in postmenopausal women, some studies observed no relationship between LDL-C level and BMD (26, 27). Moreover, Kuipers et al. (16) and Hernandez et al. (21) reported that LDL-C level was positively associated with BMD in African and Spanish men. Adami et al. (13) reported a positive relationship between LDL-C level and BMD in both healthy men and women.

An association was also found between serum cholesterol and bone turnover markers in T2D patients in our study. In men with T2D, when above the inflection point, TC and LDL-C were negatively associated with OCN, PINP, and β-CTX, and HDL level was positively associated with OCN and PTH. In women with T2D, hen above the inflection point, TC was negatively associated with OCN and β-CTX; LDL-C was inversely related to β-CTX only, while HDL-C was positively related to OCN and negatively related to PTH.

Similar findings of serum cholesterol and bone turnover markers were also reported in previous studies. Zhou et al. (28) and Chen et al. (29) found negative relationships between TC, HDL-C, LDL-C levels and OCN (28, 29). Ponda et al. reported that LDL-C was inversely correlated with serum PTH in a vitamin D repletion group (32). However, other studies found no association between serum cholesterol and bone turnover markers (12, 30, 31, 33).

**Figure 4:**
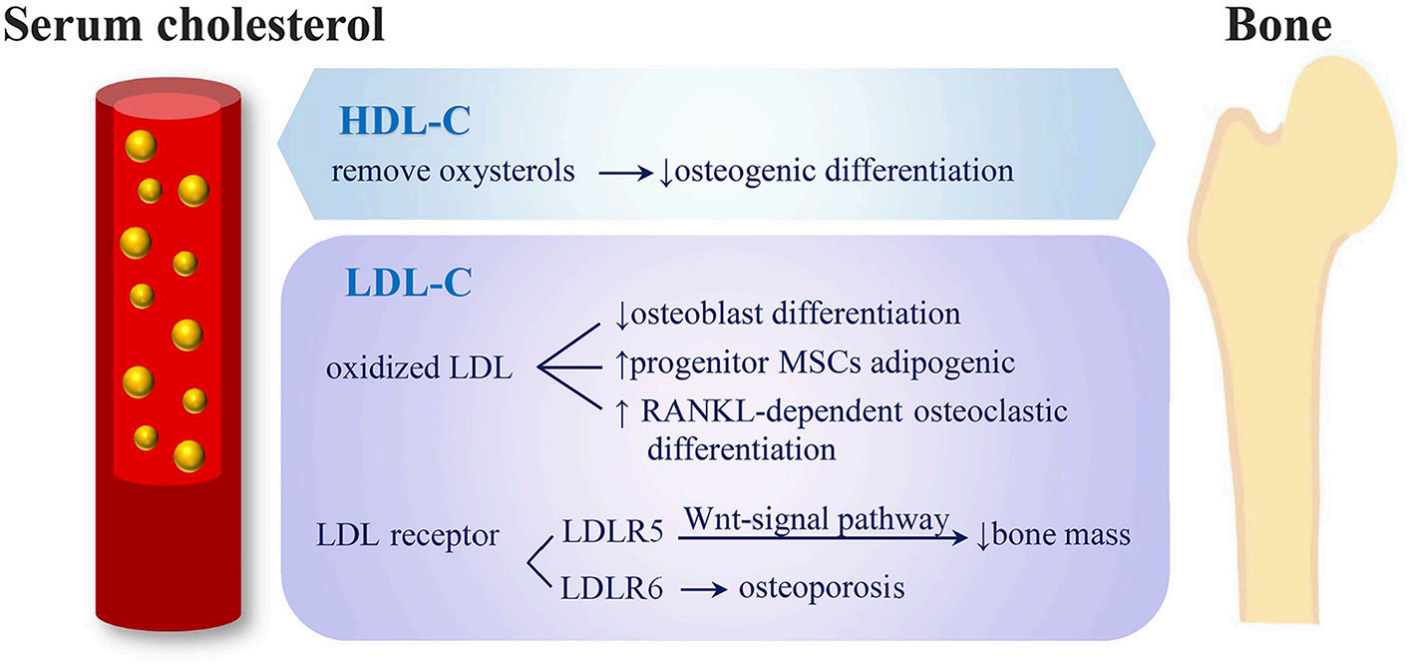
Potential mechanisms of serum cholesterol on bone metabolism. (1) HDL-C removes oxysterols from peripheral tissues and shows negative effects on osteogenic differentiation. (2) LDL oxidation products inhibit osteoblasts' differentiation, direct progenitor MSCs to undergo adipogenic instead of osteogenic differentiation and induce RANKL-dependent osteoclastic differentiation. (3) LRP5 exerts an anabolic action on bone through Wnt-signal pathway and causes low bone mass. (4) Mutation of LRP6 were related to severe osteoporosis (HDL-C, high-density lipoprotein cholesterol; LDL-C, low-density lipoprotein cholesterol; MSC, mesenchymal stem cells; RANKL, receptor activator of nuclear factor-k B ligand; LRP, low-density lipoprotein cholesterol receptor-related protein).

The potential mechanisms through which serum cholesterol may affect bone metabolism is illustrated in Figure 4. Adipocytes and osteoblasts were reported to share a common progenitor, mesenchymal stem cells (MSCs) (39, 40), and the expansion of adipose tissue in the marrow was associated with bone loss (41–43). Kha et al. (40) reported that the osteogenic differentiation of MSCs could be stimulated by specific oxysterols. Therefore, a high HDL-C level, which is able to remove oxysterols from peripheral tissues, demonstrates negative effects on osteogenic differentiation (40). Not only HDL-C level but also LDL oxidation products were reported to inhibit the differentiation of osteoblasts and direct progenitor MSCs to undergo adipogenic differentiation instead of osteogenic differentiation; thus, this reduced bone formation (44). Moreover, oxidized LDL was reported to induce receptor-activated NFκB ligand (RANKL)-dependent osteoclastic differentiation of mouse marrow preosteoclasts (45). The formation and survival of osteoclast and osteoclast activity were highly dependent on cholesterol (46, 47). LDL receptor-related protein 5 (LRP5) exerts an anabolic action on bone through the Wnt-signal pathway (48), and LRP5-deficient mice were observed to have both high cholesterol levels and low bone mass (49). In humans, LDL receptor-related protein 6 (LRP6) mutation was related to high serum LDL-C levels complicated by severe osteoporosis (50).

The effect of serum cholesterol levels on BMD may differ between cortical and trabecular bones. Experiments in mice have indicated that hyperlipidemia more prominently blunted the anabolic action of PTH in the cortical bone than in the trabecular bone (51, 52); however, Kuipers et al. (16) observed a cross-sectional association between HDL-C level and trabecular BMD but not between HDL-C level and cortical BMD or integral BMD at the hip or whole body measured by dual-energy X-ray absorptiometry (16).

According to our study, the associations between serum cholesterol and bone metabolism differed between men and women groups. We considered that these differences in results might be due to the following reasons. Firstly, the basic characteristics of the men and women groups differ. According to Table 1, men were significantly younger than women were and the diabetes durations were shorter. The mean HbA1c was higher in men, whereas, the mean β-cTX was higher in women. The proportion of smoking or drinking history was significantly higher in men than in women; and the treatment of T2D was different. These differences in characteristics between men and women groups might have led to the different BMD losses associated with increases in lipids. Secondly, in the women group, we did not consider the menopausal status or estrogen level, which might also have some effects on our study results.

Our study has several strengths. Firstly, this is the first study to report an inverse association between serum cholesterol levels and BMD in an Asian population with T2D. Secondly, our sample size was large. Thirdly, we collected complete data and adjusted for various possible confounding factors. However, our study also has several limitations. Firstly, the causal relationship between serum cholesterol levels and BMD is difficult to assess in this cross-sectional study, and our retrospective study relied on previous data. Some key statistics could not be further measured, which may affect the selection of controls. Secondly, we only collected the serum samples once from all participants, and BMD at each anatomical site was detected once; this may cause deviations in cholesterol levels and BMD values. In addition, our study used dual-energy X-ray absorptiometry to measure BMD, which may cause measurement errors and cannot distinguish between BMD of the trabecular bone and of cortical bone. Thirdly, we might have omitted some confounding variables, which might also have had some effects on our study results, such as the menopausal status, estrogen level, dietary habits, physical activity, antilipemic medication, and previous fractures.

## Conclusion

Our study suggests a significantly negative correlation between serum cholesterol levels and BMD in patients with T2D in multivariate regression analysis. The associations between serum cholesterol levels and bone turnover markers were also observed. However, further studies are required to confirm these findings.

## Author contributions

XQ and MY: designed the research. YiY, JiL, CZ, and XQ: conducted the research. GX, XY, YZ, YaY, JiL, CZ, and MY: provided the essential reagents or the essential materials. YiY, GL, and XQ: analyzed the data or performed the statistical analyses. YiY and GL: wrote the manuscript. GL, JuL, CM, XQ and MY: critical revised the manuscript.

### Conflict of interest statement

The authors declare that the research was conducted in the absence of any commercial or financial relationships that could be construed as a potential conflict of interest.

## Abbreviations

25(OH)D: 25-hydroxy-vitamin D
ALP: alkaline phosphatase
ALT: alanine aminotransferase
AST: aspartate aminotransferase
β-CTX: β-crosslaps
BMD: bone turnover marker
BMI: body mass index
BUN: blood urea nitrogen
Ca: calcium
CI: confidence interval
Cr: blood creatinine
FBG: fasting blood glucose
HbA1c: hemoglobin A1c
HDL-C: high-density lipoprotein-cholesterol
HsCRP: hypersensitive C-reactive protein
LDL-C: low-density lipoprotein-cholesterol
LRP-6: LDL receptor-related protein 6
MSC: mesenchymal stem cells
OCN: osteocalcin
PINP: procollagen type I N-terminal propeptide
PTH: parathyroid hormone
RANKL: Receptor-activated NFκB Ligand
SD: standard deviation
T2D: type 2 diabetes mellitus
TC: total cholesterol.
