# Supplementary material for: Association Between Bone Mineral Density, Bone Turnover Markers, and Serum Cholesterol Levels in Type 2 Diabetes
Source: Front Endocrinol (Lausanne). 2018 Nov 6;9:646. doi: 10.3389/fendo.2018.00646 (PMC6232230; doi:10.3389/fendo.2018.00646)
Supplement: Supplementary file 1 [file Table_1.docx]

**Table S1.** Multivariate Regression for Effect of TC, HDL-C, and LDL-C on Total Lumbar BMD (quartile division)

|  | Men | | Women | |
| --- | --- | --- | --- | --- |
|  | β(95%CI) | P | β(95%CI) | P |
| TC, mmol/l | | | | |
| <3.85 | 0 |  | 0 |  |
| >=3.85, <4.47 | -0.029 (-0.060, 0.002) | 0.07169 | -0.025 (-0.063, 0.014) | 0.21327 |
| >=4.47, <5.17 | -0.052 (-0.082, -0.022) | 0.00065 | -0.045 (-0.084, -0.005) | 0.02647 |
| >=5.17 | -0.046 (-0.076, -0.015) | 0.00347 | -0.034 (-0.074, 0.006) | 0.09670 |
| HDL-C, mmol/l | | | | |
| <0.88 | 0 |  | 0 |  |
| >=0.88, <1.05 | -0.019 (-0.050, 0.012) | 0.22810 | 0.007 (-0.033, 0.047) | 0.74333 |
| >=1.05, <1.27 | -0.052 (-0.083, -0.020) | 0.00122 | 0.004 (-0.037, 0.044) | 0.86462 |
| >=1.27 | -0.072 (-0.103, -0.040) | 0.00001 | -0.039 (-0.080, 0.003) | 0.06640 |
| LDL-C, mmol/l | | | | |
| <1.99 | 0 |  | 0 |  |
| >=1.99, <2.55 | -0.033 (-0.063, -0.002) | 0.03595 | -0.016 (-0.055, 0.023) | 0.42044 |
| >=2.55, <3.13 | -0.054 (-0.085, -0.024) | 0.00049 | -0.028 (-0.067, 0.011) | 0.16146 |
| >=3.13 | -0.048 (-0.078, -0.017) | 0.00203 | -0.032 (-0.071, 0.007) | 0.11221 |

Adjust model adjust for: Age; Diabetic duration(y); Treatment of DM; Smoking; Drinking; BMI; Cerebrovascular disease; Kidney disease; Family history of DM; Diastolic blood pressure; FBG, mmol/l; Cr, umol/l; BUN, mmol/l; Ca, mmol/l; ALT, U/L; AST, U/L; ALP, U/L
