# Supplementary material for: Association Between Bone Mineral Density, Bone Turnover Markers, and Serum Cholesterol Levels in Type 2 Diabetes
Source: Front Endocrinol (Lausanne). 2018 Nov 6;9:646. doi: 10.3389/fendo.2018.00646 (PMC6232230; doi:10.3389/fendo.2018.00646)
Supplement: Supplementary file 2 [file Table_2.docx]

**Table S2.** Multivariate Regression for Effect of TC, HDL-C, and LDL-C on Femoral Neck BMD (quartile division)

|  | Men | | Women | |
| --- | --- | --- | --- | --- |
|  | β(95%CI) | P | β(95%CI) | P |
| TC, mmol/l | | | | |
| <3.85 | 0 |  | 0 |  |
| >=3.85, <4.47 | -0.017 (-0.043, 0.010) | 0.21709 | -0.011 (-0.042, 0.020) | 0.47027 |
| >=4.47, <5.17 | -0.020 (-0.046, 0.005) | 0.12215 | -0.020 (-0.051, 0.012) | 0.21882 |
| >=5.17 | -0.025 (-0.051, 0.001) | 0.05805 | -0.021 (-0.053, 0.011) | 0.19943 |
| HDL-C, mmol/l | | | | |
| <0.88 | 0 |  | 0 |  |
| >=0.88, <1.05 | -0.0014 (-0.040, 0.012) | 0.28854 | -0.020 (-0.052, 0.012) | 0.21349 |
| >=1.05, <1.27 | -0.030 (-0.056, -0.004) | 0.02654 | -0.007 (-0.040, 0.025) | 0.65883 |
| >=1.27 | -0.065 (-0.092, -0.038) | <0.00001 | -0.041 (-0.074, -0.008) | 0.01493 |
| LDL-C, mmol/l | | | | |
| <1.99 | 0 |  | 0 |  |
| >=1.99, <2.55 | -0.008 (-0.034, 0.017) | 0.51994 | -0.023 (-0.054, 0.008) | 0.15326 |
| >=2.55, <3.13 | -0.022 (-0.048, 0.004) | 0.09736 | -0.032 (-0.063, -0.001) | 0.04088 |
| >=3.13 | -0.020 (-0.046, 0.006) | 0.12509 | -0.033 (-0.064, -0.002) | 0.03879 |

Adjust model adjust for: Age; Diabetic duration(y); Treatment of DM; Smoking; Drinking; BMI; Cerebrovascular disease; Kidney disease; Family history of DM; Diastolic blood pressure; FBG, mmol/l; Cr, umol/l; BUN, mmol/l; Ca, mmol/l; ALT, U/L; AST, U/L; ALP, U/L
