# Supplementary material for: Association Between Bone Mineral Density, Bone Turnover Markers, and Serum Cholesterol Levels in Type 2 Diabetes
Source: Front Endocrinol (Lausanne). 2018 Nov 6;9:646. doi: 10.3389/fendo.2018.00646 (PMC6232230; doi:10.3389/fendo.2018.00646)
Supplement: Supplementary file 3 [file Table_3.docx]

**Table S3.** Multivariate Regression for Effect of TC, HDL-C, and LDL-C on Total Hip BMD (quartile division)

|  | Men | | Women | |
| --- | --- | --- | --- | --- |
|  | β(95%CI) | P | β(95%CI) | P |
| TC, mmol/l | | | | |
| <3.85 | 0 |  | 0 |  |
| >=3.85, <4.47 | -0.005 (-0.032, 0.023) | 0.74784 | -0.019 (-0.052, 0.015) | 0.27349 |
| >=4.47, <5.17 | -0.009 (-0.036, 0.017) | 0.48806 | -0.024 (-0.058, 0.010) | 0.17502 |
| >=5.17 | -0.020 (-0.048, 0.007) | 0.14369 | -0.022 (-0.057, 0.012) | 0.20562 |
| HDL-C, mmol/l | | | | |
| <0.88 | 0 |  | 0 |  |
| >=0.88, <1.05 | -0.008 (-0.035, 0.019) | 0.56035 | -0.022 (-0.056, 0.011) | 0.19444 |
| >=1.05, <1.27 | -0.024 (-0.051, 0.004) | 0.09020 | -0.018 (-0.053, 0.017) | 0.31177 |
| >=1.27 | -0.069 (-0.096, -0.041) | <0.00001 | -0.062 (-0.097, -0.027) | 0.00064 |
| LDL-C, mmol/l | | | | |
| <1.99 | 0 |  | 0 |  |
| >=1.99, <2.55 | 0.009 (-0.018, 0.036) | 0.50866 | -0.025 (-0.059, 0.009) | 0.14694 |
| >=2.55, <3.13 | -0.005 (-0.032, 0.022) | 0.72983 | -0.024 (-0.058, 0.009) | 0.15629 |
| >=3.13 | -0.017 (-0.044, 0.010) | 0.21539 | -0.033 (-0.067, 0.001) | 0.05792 |

Adjust model adjust for: Age; Diabetic duration(y); Treatment of DM; Smoking; Drinking; BMI; Cerebrovascular disease; Kidney disease; Family history of DM; Diastolic blood pressure; FBG, mmol/l; Cr, umol/l; BUN, mmol/l; Ca, mmol/l; ALT, U/L; AST, U/L; ALP, U/L
